# Supplementary figures and images for: Links between Soil Bacteriobiomes and Fungistasis toward Fungi Infecting the Colorado Potato Beetle
Source: Microorganisms. 2023 Apr 4;11(4):943. doi: 10.3390/microorganisms11040943 (PMC10141481; doi:10.3390/microorganisms11040943)

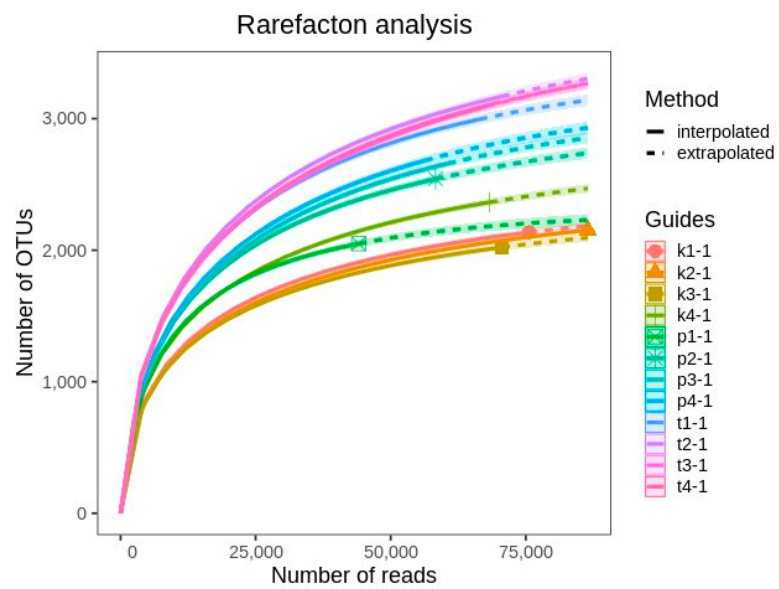

Figure S1. Rarefaction analysis of the investigated samples. k: Karasuk, p: Novosiborsk, t: Toguchin.

Supplement: Supplementary file 1 [file microorganisms-11-00943-s001.zip › Figure S1.pdf]
